# The impact of cryopreservation on cytokine secretion and polyfunctionality in human PBMCs: a comparative study

**DOI:** 10.3389/fimmu.2024.1478311

**Published:** 2024-10-07

**Authors:** Aline Linder, Kevin Portmann, Klaus Eyer

**Affiliations:** ^1^ Laboratory for Functional Immune Repertoire Analysis, Institute of Pharmaceutical Sciences, Department of Chemistry and Applied Biosciences, ETH Zurich, Zurich, Switzerland; ^2^ Department of Biomedicine, Aarhus University, Aarhus, Denmark

**Keywords:** human peripheral blood mononuclear cells, single-cell analysis, cytokine secretion, polyfunctionality, cryopreservation

## Abstract

**Introduction:**

Human peripheral blood mononuclear cells (hPBMCs) are widely used in fundamental research and clinical applications as studying their responses to *in vitro* activation is an effective way to uncover functional alterations and disease associated phenotypes. However, the availability of samples in large numbers at a specific time and location remains challenging, hence they often might preferably be collected and cryopreserved for later analysis. While the effect of cryopreservation on viability and cell surface expression is well established, changes in activity and cytokine secretion still lead to conflicting results as it is often measured in bulk or within the cells.

**Methods:**

Here, we used our platform for dynamic single-cell multiplexed cytokine secretion measurement and compared it to a traditional intracellular cytokine staining to quantify the effect of cryopreservation on cytokine secretion and expression of individual hPBMCs.

**Results:**

Following stimulation with LPS or anti-CD3/CD28 antibodies for up to 36 or 72 h incubation, we observed distinct alterations in cytokine responses due to cryopreservation when comparing to fresh samples, but also remarkable consistencies for some cytokines and parameters. In short, the frequencies of cytokine-secreting cells in cryopreserved samples were lower for IL-6 (LPS), IL1-β (CD3/CD28) and IFN-γ (CD3/CD28), while the frequency and dynamics of IL-8 secretion were strongly impacted in all cases. We observed a large disconnect between cytokine expression and secretion for TNF-α, where the expression dramatically increased after cryopreservation, but actual secretion was, in comparison, remarkably stable. The polyfunctionality of single cells was altered by cryopreservation in specific co-secreting populations led by the effects on IL-6 or IL-8 secretion. Among immune cells, cryopreservation seemed to affect lymphocytes and monocytes differently as effects appeared early on in lymphocytes while generally observed in later time points in monocytes.

**Conclusion:**

Together, this study offers an in-depth quantitative insight into the biological behavior of immune cells in response to cryopreservation and stimulation, further providing some insights into conflicting results in the literature as well as guidelines for researchers planning to assess cytokine-secreting from frozen hPBMCs in immunological research or clinical applications.

## Introduction

Human peripheral blood mononuclear cells (hPBMCs) and their isolated cellular subpopulations are essential tools in immunological research and beyond. hPBMCs are widely utilized to model various immune disorders and to develop and test candidate drugs and vaccines (1, 2). Extending toward clinical applications, recent scientific studies using hPBMCs have been focusing on establishing new biomarkers for autoimmune diseases to assist in diagnosis and evaluate therapeutic efficacy (3–5). These scientific studies encompassed a range of approaches, including genomic profiling, transcriptomic analysis, metabolomics, proteomics, and the analysis of specific cellular subpopulations (5–9). Furthermore, hPBMCs are integral to advanced therapeutic strategies, such as the generation of chimeric antigen receptor (CAR) T cells for subsequent treatments (10, 11). Consequently, hPBMCs are indispensable in research, preclinical studies, and clinical applications as a versatile source material.

Cryopreservation often remains an inevitable step in studying hPBMC phenotypes and functionalities, especially in the context of rare diseases where samples are scarce and difficult to obtain centrally. Cryopreservation is also crucial for establishing biobanks and enabling the study of historical samples with new methodologies. Maintaining the functional capacity and viability of these cells is especially vital when they are intended for diagnostic or therapeutic purposes. Indeed, without a reliable cryopreservation protocol, detrimental batch effects could occur during the serial measurements of large cohort of samples and lead to misleading outcomes, especially in studies where time is confounded with batch. While cryopreservation has been shown to have minimal effects on cell viability and surface markers if done properly (12, 13), changes in cellular composition and conflicting results regarding secreted cytokines have been reported (14–16).

Cytokines and other secreted proteins are closely associated with immune activation and status (13), playing an important role in the pathogenesis of various diseases by serving as key immune messengers between cells. As such, they offer valuable insights into disease progression and state (17). For example, differential cytokine secretion profiles have been linked to immune complications of viral infections and adverse reactions during immunotherapy, highlighting their potential in biomarker discovery (18–20). Typically, cytokine secretion is reported as concentration levels in the supernatant or through intracellular cytokine staining (ICS) to estimate cytokine production and the frequency of expressing cells (21–23). Other commonly employed methods include methods like ELISpot and microchamber/microarray measurements (24–26).

Indeed, a reduced cytokine concentration measured in bulk, for example, could have several origins, such as a general reduction of secretion, the functional inactivity of a specific cell population, delayed secretion dynamics, or a combination of these points. None of the aforementioned techniques enable real-time, dynamic, and quantitative secretion measurements with single-cell resolution, making it difficult to accurately link observed changes to specific biological mechanisms. Therefore, we identified the lack of dynamic, functional, and quantitative single-cell data as a gap in the current literature that studies or utilizes cryopreserved hPBMCs.

**Figure 1:**
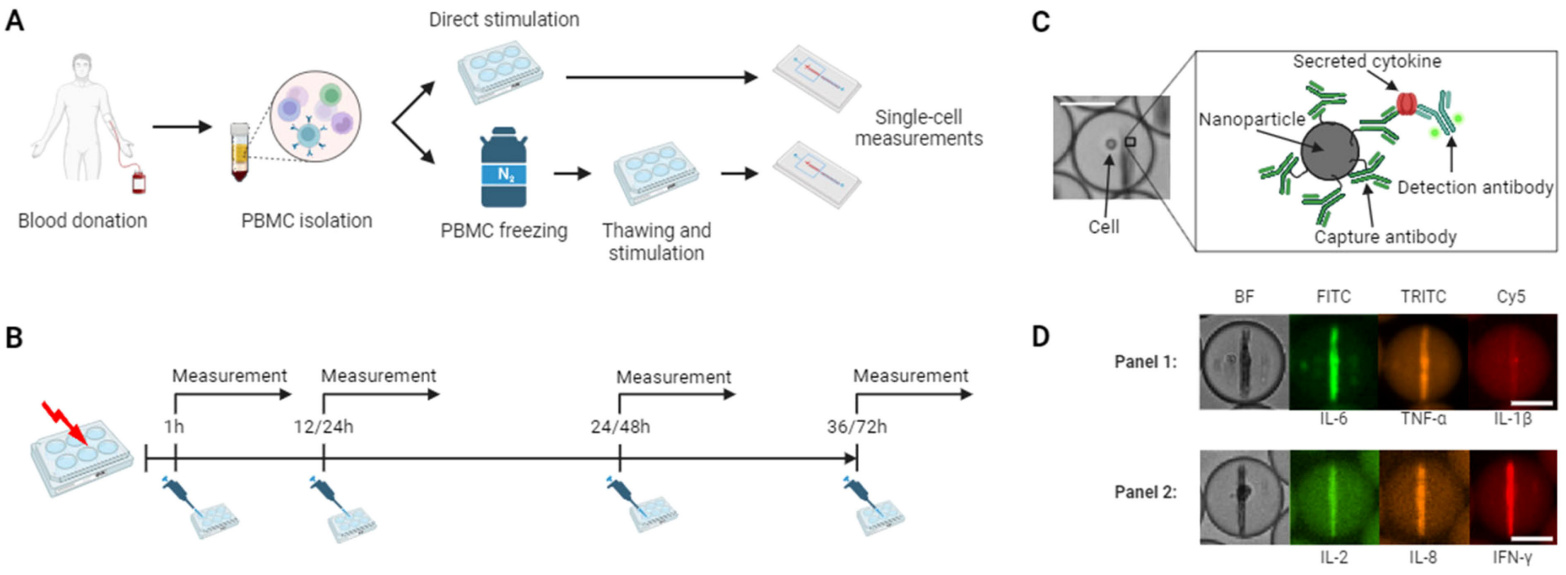
Workflow of stimulation and cytokine secretion experiments. **(A)** Experimental protocol for hPBMCs isolation and stimulation. The cells were isolated through density gradient centrifugation from healthy donors, half of the cells were directly stimulated and subjected to single-cell measurements, while the other half was frozen, stored in liquid nitrogen, followed by thawing, stimulation, and single-cell measurements. **(B)** Stimulation protocol. Fresh and cryopreserved cells were stimulated in bulk with lipopolysaccharide (LPS) or anti-CD3/CD28 antibodies and incubated with subsequent microfluidic measurements after 1, 12, 24 and 36 hours or 1, 14, 48 and 72 hours, respectively. **(C)** Principle of the in-droplet sandwich immunoassay used for cytokine detection. **(D)** Using differently functionalized nanoparticles and fluorescent antibodies, simultaneous measurements of up to three cytokines were possible in two separate panels consisting of IL-6, TNF-α, IL-1β and IL-2, IL-8, IFN-γ were measured in this study for each cell. The hPBMCs were encapsulated with assay reagents into 65 picoliter water-in-oil droplets, immobilized and imaged every 30 minutes for 4 hours. The chosen images depict each one cell secreting either IL-6^+^/TNF-α^+^/IL-1β^-^ and IL-2^+^/IL-8^+^/IFN-γ^+^. Scale bars: 25 μm. **(A-C)** were created with BioRender.com.

To resolve these parameters, we have developed a dynamic multiplexed cytokine secretion method that enables highly sensitive and resolved measurements of individual cytokine-secreting cells (27, 28) (**Figure 1**). In this approach, single cells are encapsulated in water-in-oil emulsions, allowing the quantitative and dynamic assessment of secretion of up to three cytokines using specific sandwich immune assays. Time-resolved fluorescence microscopy further facilitates the readout of individual secretion dynamics and quantification of each cytokine at multiple time points, generating a high-resolution image of cytokine secretion on the single-cell level (29). In this study, we applied this platform to examine the effect of cryopreservation on differential cytokine secretion from bulk-stimulated hPBMCs, comparing the results to traditional intracellular cytokine staining. Herein, we investigated the impact of cryopreservation on the secretion dynamics of cytokines IL-6, TNF-α, IL-1β, IL-2, IL-8, and IFN-γ across various stimulants and time points. Our analysis focused on secreted amounts per cell, frequencies of secreting cells, polyfunctional cells, and other potential time-dependent immune activation processes that may be altered post-freezing. This approach generated a highly quantitative, dynamically resolved dataset, providing new insights into the effects of cryopreservation on PBMC functionality.

## Materials and methods

### Cell isolation and handling

hPBMCs were isolated from a buffy coat obtained from the Zurich blood bank using density gradient centrifugation. All experiments were conducted with hPBMCs obtained from anonymous, healthy donors, in agreement with ethics agreement EK 202-N-56, approved by the ETH Zurich ethics commission. The buffy coats were diluted 1:1 in DPBS (-Ca/-Mg, ThermoFisher) with added 2 mM EDTA (Sigma-Aldrich), and centrifuged at 1200 g for 20 minutes over a Ficoll layer (GE Healthcare). Afterward, the hPBMCs were collected, washed with DPBS/EDTA and the red blood cells lysed (BDPharmLyse, BD Biosciences) for 5 min, RT. Following lysis, the cells were washed with phenol red-free completed RPMI 1640 medium containing 10% heat-inactivated FBS, 25 mM HEPES, and 50 U/ml Pen/Strep (all ThermoFisher). The hPBMCs were then counted and divided for the respective frozen and fresh measurements. The cells designated to cryopreservation were washed with completed RPMI, aliquoted in qualified heat-inactivated FBS (ThermoFisher) containing 10% DMSO (Applichem) at 10^7^ cells/vial, subsequently frozen overnight at -80°C using a Mr. Frosty**™** freezing container (ThermoFisher). The next day, the vials were transferred into liquid nitrogen. The cells were stored in liquid nitrogen for at least 2 days and a maximum of 19 days. The frozen hPBMCs samples were thawed at 37°C in completed RPMI and washed two times in completed RPMI. Three different buffy coats were used: in the microfluidic experiment for LPS (i) and anti-CD3/CD28 stimulation (ii), and in the intracellular cytokine staining for both stimulations (iii). In each experiment, the same buffy coat was used to allow for direct comparison within the condition of fresh and frozen cells. Further information on buffy coat batches, blood volumes, cell viability and counts linked to the experiments are described in **Table 1**.

**Table 1:** hPBMCs count and viability after fresh blood processing and post thawing.

	Buffy coat batch	1	2	3
	Experiment	Microfluidic	Microfluidic	ICS
	Condition	anti-CD3/CD28 antibodies	LPS	LPS, anti-CD3/CD28 antibodies and unstimulated
	Volume of blood received approx. of buffy coat	50ml	50ml	50ml
After fresh blood processing	Cell viability [%]	99	96	96
	Total cell count [x10^7^]	47.8	113	50.0
Fresh hPBMCs, after staining/before stimulation (Microfluidic)	Cell viability [%]	98	97	
	Total cell count [x10^6^]	18.2	14.1	
Post thawing	Cell viability [%]	92	93	82
	Total cell count [x10^6^]	18.7	14.0	149
Thawed hPBMCs, after staining/before stimulation (Microfluidic)	Cell viability [%]	93	92	
	Total cell count [x10^6^]	12.8	9.2	

### Cell staining for microfluidic experiments

The cells were resuspended to a final concentration of 10^6^ cells/ml and stained with 5 µM CellTrace violet (ThermoFisher) and FcR-blocked with human FcR blocking reagent (Miltenyi Biotec, 1:4 dilution) in DPBS. After blocking, the hPBMCs were washed and resuspended to a final concentration of 10^6^ cells/ml.

### Cell stimulation for microfluidic experiments

A total of 2x10^6^ hPBMCs, either fresh or thawed, were stimulated with either 1 µg/ml lipopolysaccharide (LPS, Invivogen) for 1, 12, 24 and 36 hours, or with 5 µg/ml anti-CD3 (OKT3, ThermoFisher) and 5 µg/ml anti-CD28 (CD28.2, ThermoFisher) for 1, 24, 48 and 72 hours at 37°C and 5% CO_2_. The experiments were performed in ultra-low attachment 6-well plates (Corning), with wells thoroughly scraped after incubation to detach all hPBMCs. The cells were washed two times after incubation and resuspended in completed RPMI.

### Solutions for microfluidic experiments

The protocol of nanoparticle functionalization has been described in great detail in the literature (29). In this study, two panels measuring IL-6/TNF-α/IL-1β and IL-2/IL-8/IFN-γ were used. For this, individual nanoparticle solutions against IL-6/TNF-α/IL-1β were mixed at a 2:1:1 ratio, and for IL-2/IL-8/IFN-γ particles were mixed 1.66:1.33:1 ratio, resulting in each case in 2’400 particles per droplet. Information about the used capture and detection antibodies, as well as used concentrations and limit-of-detection of the assay, can be found elsewhere (27). Final cell concentrations for microfluidic cell encapsulations were 8 – 16 x 10^6^ cells/ml, achieving cell/droplet ratios of 0.2 – 0.4.

### Observation chamber assembly, PDMS chip production and droplet generation

The observation chamber was produced using methods described elsewhere (29), allowing for the production of droplets using a hydrodynamic flow-focusing PDMS chip, following methods previously described (30).

### Data analysis of microfluidic experiments

In brief, fluorescence is read out for every droplet on the nanoparticles and in the background of the droplet via a custom Matlab script (Mathworks, version R2020A). With this, fluorescence relocation onto the nanoparticles can be calculated for every individual droplet and time point. Cytokine-secreting cells are then identified based on a set of different criteria: Droplet contained an encapsulated cell (Identified through a positive DAPI signal), the fluorescence relocation increases over the measurement time (slope > 0), the maximum beadline relocation is above the LOD of the measurement, calculated according to the following formula:


$$\mu_{Relocation\;t0}+1.645\times 2\times \;\sigma_{BLK},$$

and the change between maximum and minimum fluorescence relocation is bigger than:


$$1.645\times 2\times \;\sigma_{BLK}$$

The fluorescence relocation of the positively identified droplets is then quantified using calibration curves specific for the measured channel and cytokine panel (**Supplementary Figure S1**), and subsequently calculated into secretion rates via the overtime concentration changes. If a droplet reached the maximum quantifiable relocation before the end of the experiment, the concentration was set to the maximum, and no further concentration was calculated. The secretion rate was only averaged until this time point. The standard deviation of the blank was acquired from calibration data sets. If a cell met criteria in more than one channel, it was deemed a co-secreting or polyfunctional cell. For the percentage of secreting cells, the total cell count was determined by using another custom Matlab script, requiring manual analysis of around 500 droplets per measurement. For more information, see (27). Whenever less than 50 droplets met the secretion criteria, they were analyzed manually to exclude potential fluorescence aggregates or improper aggregation of the nanoparticles. If less than 10 events were identified as positive, they were not considered for analysis.

### Calibration curves

Instead of hPBMCs in complete RPMI, recombinant proteins were used to calibrate the relocation signal to concentration. The fluorescence relocation against cytokine concentration was fitted with a one-phase association curve fit with GraphPad Prism (**Supplementary Figure S1**), and the resulting equations were used to quantify the cellular secretion data. For more information about used proteins and control experiments, please refer to Portmann et al. (27).

### Intracellular cytokine staining and multiparametric flow cytometry

After isolation or thawing, 10^6^ cells per well (150 µl) were stimulated with 1 µg/ml lipopolysaccharide (LPS, Invivogen) or 5 µg/ml anti-CD3 (OKT3, ThermoFisher) + 5 µg/ml anti-CD28 (CD28.2, ThermoFisher) antibodies for 1 and 24 hours. At the end of the stimulation time, 10 µg/ml Brefeldin A (Sigma-Adrich) and 0.7 µl/ml GolgiStop (BD Biosciences) were added and the cells incubated for another 4 hours at 37°C, which represented the stimulation and measurement time of the droplet experiments. After, the cells were washed twice with 200 μl/well PBS (ThermoFisher, 10010-015), and the dead cells were stained with 100 μl/well of eBioscience™ Fixable Viability Dye eFluor™ 450 (ThermoFisher, diluted 1:3’000 in PBS) for 10 min at RT. The cells were washed once in PBS and once in eBioscience™ Flow Cytometry Staining Buffer (ThermoFisher). Afterward, the hPBMCs were blocked using 50 μl/well of human FcR block (Miltenyi Biotec) diluted at 1:25 for 10 min at RT. Surface staining was performed by adding 50 μl/well of diluted antibodies (see **Table 2**) in Flow Cytometry Staining Buffer for 30 min at 4°C. The hPBMCs were then washed twice with Flow Cytometry Staining Buffer, fixed with Fixation/Permeabilization solution (BD Biosciences) for 20 min at 4°C and permeabilized through two washes with Perm/Wash Buffer (BD Biosciences). Intracellular staining was then performed by adding 50 μl/well of diluted antibody (**Table 2**) in Perm/Wash Buffer at 4°C for 30 min. The cells were washed twice with Perm/Wash Buffer and resuspended in 100 μl/well Flow Cytometry Staining Buffer. The samples were run on a CytoFLEX S (Beckman Coulter) and analyzed using FlowJo software version 10.8.1 (BD Life Sciences). The gating strategy is presented in **Supplementary Figure S2**. Identified positive events are shown as a percentage in relation to all alive cells, or as a relative percentage of the parent gate (for subpopulations). Stained samples were analyzed using appropriate compensation for correcting spectral overlap and autofluorescence.

**Table 2:** Used antibodies for cell surface and intracellular cytokine staining (ICS).

Reagents	Supplier	Catalog number
Extracellular staining		
CD8a Monoclonal Antibody (RPA-T8), PerCP-Cyanine5.5	ThermoFisher	45-0088-41
CD16 Monoclonal Antibody (eBioCB16 (CB16)), Alexa Fluor™ 700	ThermoFisher	56-0168-42
CD14 Monoclonal Antibody (61D3), APC-eFluor™ 780	ThermoFisher	47-0149-41
CD3 Monoclonal Antibody (UCHT1), eFluor™ 506	ThermoFisher	69-0038-41
CD4 Monoclonal Antibody (RPA-T4), Super Bright™ 600	ThermoFisher	63-0049-41
BV650 Mouse Anti-Human CD56	BD Biosciences	564057
Super Bright Complete Staining Buffer	ThermoFisher	SB-4401-42
CellBlox™ Blocking Buffer	ThermoFisher	B001T03F01
Intracellular staining		
IL-6 Monoclonal Antibody (MQ2-13A5), FITC	ThermoFisher	11-7069-81
IL-2 Monoclonal Antibody (MQ1-17H12), FITC	ThermoFisher	11-7029-42
IL-1β (Monoclonal Mouse) in house labeled with AF647	Peprotech	500-M01B
IFN gamma Monoclonal Antibody (4S.B3), APC	ThermoFisher	17-7319-82
TNF alpha Monoclonal Antibody (MAb11), PE	ThermoFisher	12-7349-81
Anti-Human IL-8 (CXCL8), in house labeled with AF555	Peprotech	500-M08

### Quantification and statistical analysis

Replicates are presented as mean ± SEM or SD, where n refers to the number of biological replicates. Differences in distributions were assessed using two-sided, unpaired, non-parametric Kolmogorov-Smirnov t-tests. Differences in the average percentage of positive cells were assessed using multiple unpaired Welch t-tests with an alpha of 0.05 (Holm-Šídák method). p-values were denoted as follows: * < 0.05, ** < 0.01, *** < 0.001, **** < 0.0001.

## Results

### After LPS stimulation, TNF-α and IL-6 SC displayed lower frequencies, IL-1β secretion rates were lower in longer incubation times while IL-8 secretion was strongly affected by cryopreservation

**Figure 2:**
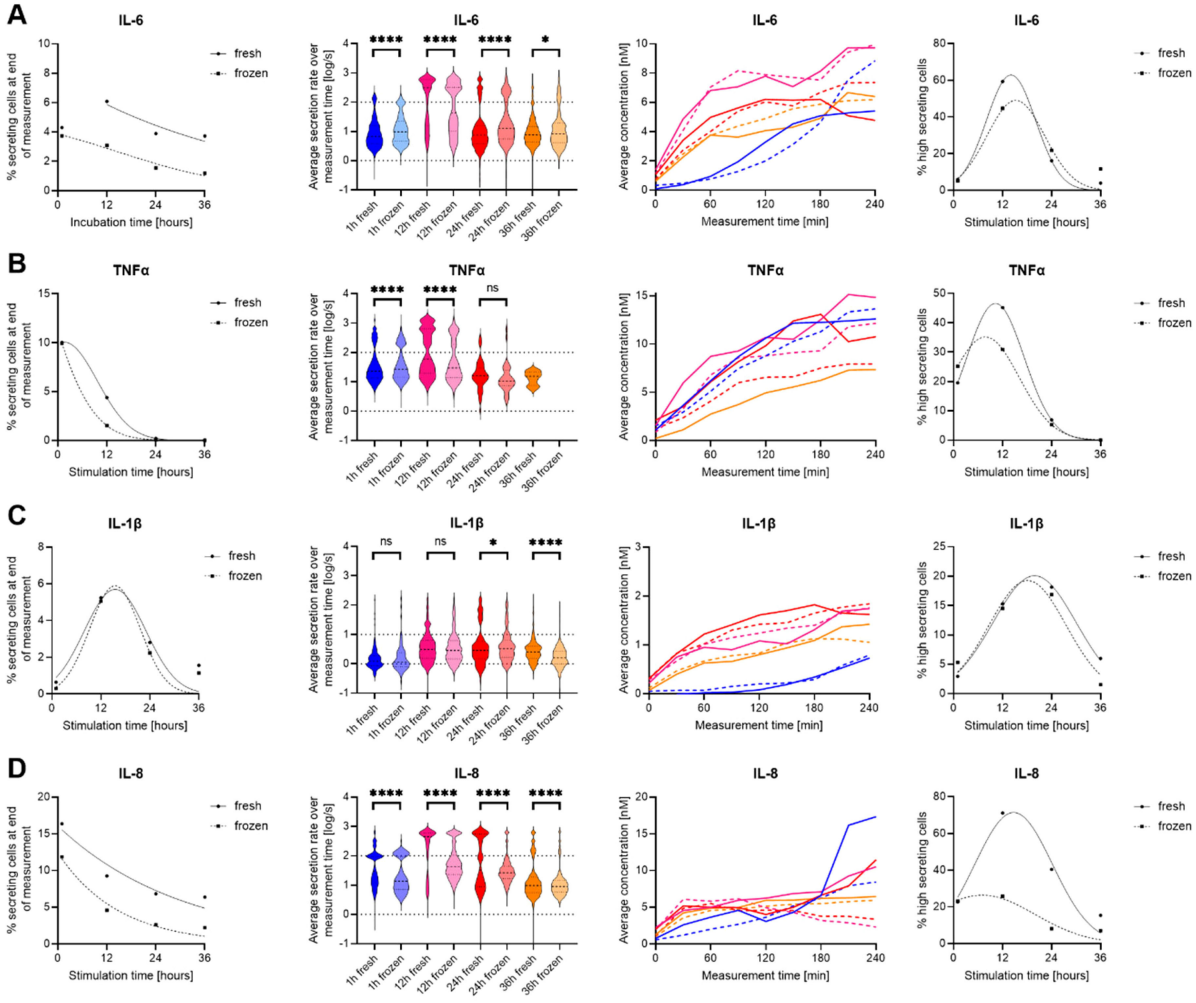
Differences in secretion amounts and dynamics IL-6, TNF-α, IL-1β and IL-8 secretion of fresh and cryopreserved LPS stimulated PBMC. Data is shown for IL-6 **(A)**, TNF-α **(B)**, IL-1β **(C)** and IL-8 **(D)**, while no secretion was measured for IL-2 and IFN-γ. The panels, in order from left to right, show the percentage of cytokine-secreting cells at the different measurement times (1^st^ panel), the over-time averaged secretion rate (log) distributions (2^nd^ panel), the average concentration secreted over time of all secreting cells that did not reach the assay plateau (3^rd^ panel), and the percentage of high-secreting cells (>100 molecules/s for IL-6, TNF-α, IL-8 and >10 molecules/s for IL-1β (4^th^ panel). In panels 1, 3 and 4, fresh (cont. lines) and frozen (dotted lines) PBMC were plotted, and in panels 2 and 3, the data was color-coded for 1 h (blue), 12 h (pink), 24 (red) and 36 h (orange) stimulated fresh (cont. lines) or frozen (dotted lines) hPBMCs. Panels 1 and 4 were fitted with Gaussian distribution fits for visualization (GraphPad Prism), and data with less than ten detected secreting cells were not plotted. Statistical differences in secretion rate distributions were assessed using two-sided, unpaired, non-parametric Kolmogorov-Smirnov t-tests with 95% confidence and p < 0.05. p-values were denoted as follows: * < 0.05, ** < 0.01, *** < 0.001, **** < 0.0001. ns refers to non-signficant.

To investigate the potential influence of cryopreservation on the dynamics of cytokine secretion and polyfunctionality of innate immune cells especially, we compared freshly isolated to cryopreserved hPBMCs from the same donor in response to lipopolysaccharide (LPS) stimulations (31). To achieve further dynamic resolution, as freezing might also shift the time needed to respond with secretion, we measured the secretion amounts and dynamics of IL-6, TNF-α, IL-1β, IL-2, IL-8, and IFN-γ after 1, 12, 24 and 48 h stimulation times. As expected with this stimulant, we observed secretion for IL-6, TNF-α, IL-1β and IL-8 after LPS stimulation (**Figure 2**), and no secretion of IL-2 and IFN-γ.

Starting with IL-6, the percentage of IL-6 secreting cells (SC) decreased with prolonged stimulation times for fresh and cryopreserved hPBMCs (**Figure 2A**). Generally, the frozen cells displayed a reduction in SC of 2-3% for every later time point, while this difference was not present early on at the one-hour stimulation (4.3% *vs.* 3.7%, respectively). On the single-cell secretion levels (**Figure 2A**, 2^nd^ panel), the secretion rate distributions showed a highly significant difference for 1-, 12- and 24-hours of stimulation (p < 0.0001 for all) and resolved to a slight difference for the 36-hour stimulation (p = 0.04). While thawed cells secreted slightly more IL-6, the average single-cell secretion distribution showed the appearance of two clear populations for low- and high-secreting cells, which appeared at 12h and disappeared progressively over time in both fresh and frozen cells. This consistency was also apparent in the 3^rd^ and 4^th^ panels, where only minor differences were visible. Therefore, while the percentages of IL-6 SC were different at longer incubation time points and the single-cell distributions displayed significant differences, the overall secretion dynamics were very comparable.

For TNF-α SC, the observed percentage of secreting cells started at similar levels (10%) but rapidly decreased to almost 0% for both over the assayed stimulation period (**Figure 2B**). This decrease was faster in thawed cells as a strong difference between fresh and frozen cells was observed after 12 hours of stimulation (4.4% *vs.* 1.5%). We detected only very few TNF-α SC after 36 h stimulation, only 1 in frozen hPBMCs compared to 16 in fresh cells. Interestingly, the secretion rate distributions however showed a significant difference already at the 1-hour analysis and was still present at 12-hour stimulated cells (p < 0.0001 both), but the difference vanished after 24 h of stimulation (p > 0.05) likely due to the low number of secreting cells detected in the later timepoints. The differences in the secretion rate distributions were mostly due to different numbers of high-SC, where the fresh SC showed a smaller fraction at 1 h that increased at 12 h (19.5% to 45.2%) whereas it was seen to a lesser extent in thawed cells (25.20 to 30.9%, 4^th^ panel). In summary, the secretion dynamics were very similar for TNF-α SC, and interestingly the secretion responses of TNF-α and IL-6 were alike, potentially indicating similar cells and pathways being influenced by the freezing and thawing process.

In contrast to IL-6 and TNF-α, IL-1β secretion was not found to be heavily influenced by cryopreservation (**Figure 2C**). The percentages of SC were very similar over different stimulation times, and the secretion dynamics did not show considerate change when comparing fresh to frozen cells. On the single-cell level, IL-1β was again very similar, with the only differences at prolonged incubation times, observed to increase from 24- (p = 0.04) to 36 h of stimulation (p < 0.0001), where freshly isolated and stimulated cells had higher secretion rates of IL-1β. However, in this case, the percentage of high IL-1β SC (>10 molecules/s) did not show considerate differences for fresh and cryopreserved cells. Therefore, IL-1β secretion was not affected by cryopreservation with a modification in the frequency of secreting cells.

Lastly, IL-8 SC displayed the largest susceptibility to cryopreservation (**Figure 2D**), with considerably lower percentages of SC over all the measured stimulation times for frozen cells. In general, around 5% less IL-8 SC were observed at all investigated time points. Also, the secretion rate distributions showed highly significant differences for all stimulation time points (p < 0.0001), and while the whole population of SC was affected after freezing, mainly high IL-8 secreting cells were drastically reduced (**Figure 2D**, 4^th^ panel). Indeed, the loss of high-secreting cells was predominantly apparent after 12- (71.2% vs. 25.9%) and 24-hour stimulations (40.4% vs. 8.2%). Therefore, all assayed parameters and time points for IL-8 were affected by the freeze-thawing process prior to stimulation.

### Stimulation with anti-CD3/CD28 antibodies led to increased IL-2 and IL-8, and decreased IFN-γ, TNF-α, IL-1β SC in frozen hPBMCs

As hPBMCs are a heterogeneous cell population, we expended the used stimulants to an anti-CD3/CD28 antibody cocktail to investigate potential influences of the cryopreservation process in adaptive immune cell subsets, specifically T cells (32). Since previously published results showed a slower response dynamic (27), we increased the measured stimulation times to 1, 24, 48 and 72 h. In these assays, all six measured cytokines were at least partly secreted.

**Figure 3:**
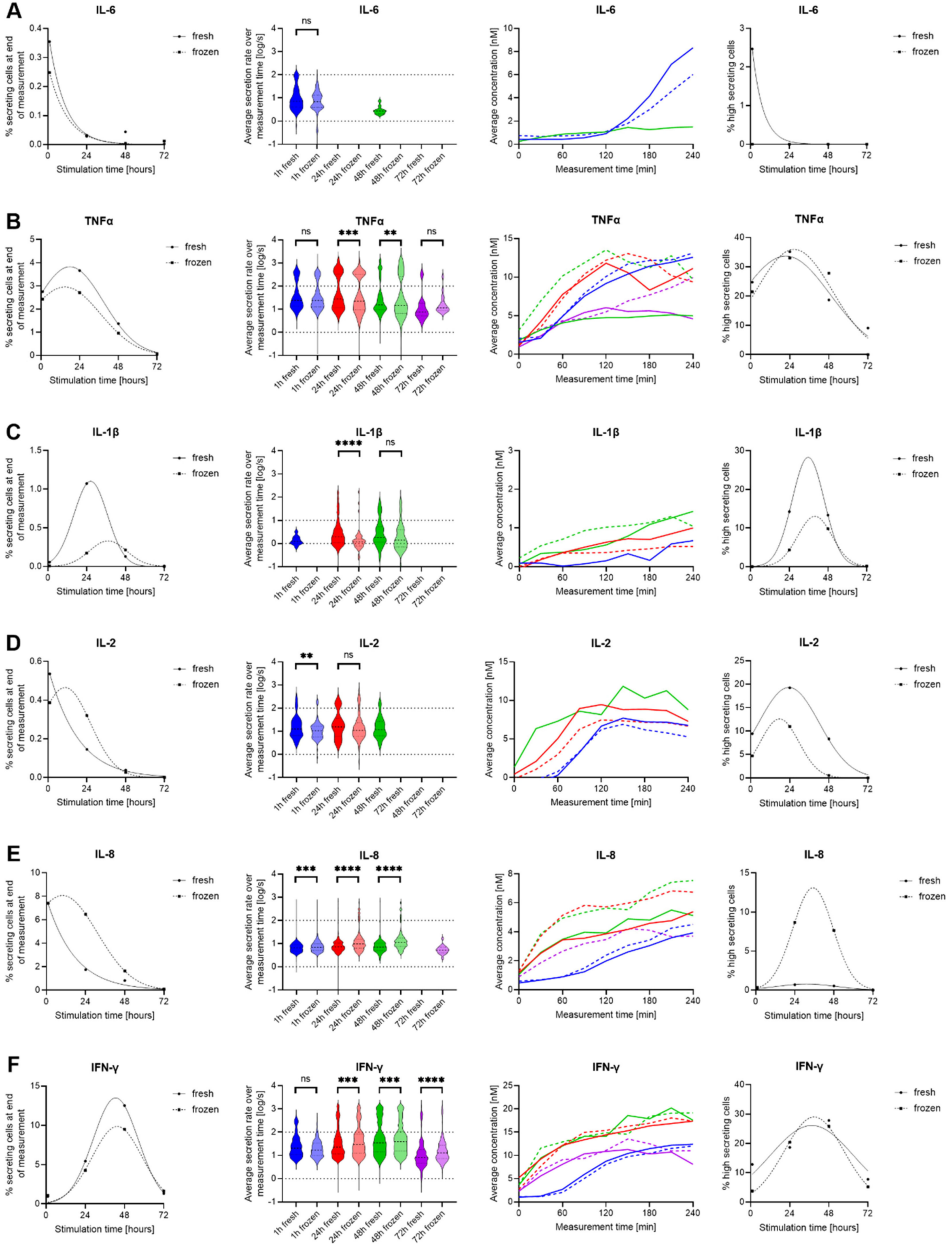
Differences in IL-6, TNF-α, IL-1β, IL-8, IL-2 and IFN-γ secretion of fresh and cryopreserved anti-CD3/CD28 stimulated hPBMCs. IL-6 **(A)**, TNF-α **(B)**, IL-1β **(C)**, IL-8 **(D)**, IL-2 **(E)** and IFN-γ **(F)** were measured following stimulation for 1- (blue), 12- (red), 24- (green) and 36-hour (purple) in fresh (cont. lines) or frozen (dotted lines) PBMC. The data is displayed as described in the caption of **Figure 2**. p-values were denoted as follows: * < 0.05, ** < 0.01, *** < 0.001, **** < 0.0001.

In contrast to LPS stimulation, the activation of hPBMCs with anti-CD3/CD28 antibodies only resulted in a very minor activation of IL-6 SC (**Figure 3A**). Low frequencies were detected across all measurements, with no observed differences in secretion dynamics or rates (p = 0.6) between fresh and thawed stimulated hPBMCs.

Contrary to IL-6, TNF-α SC were present after anti-CD3/CD28 stimulation at all time points, namely 1-, 24-, 48- and 72-hour stimulations, with slightly higher frequencies for freshly isolated hPBMCs (**Figure 3B**). Significant alterations in secretion rate distributions were observed after 24 hours (p=0.0005) and after 48 hours of stimulations (p = 0.002), whereas early and late time points were similar. This difference was due to an increased appearance of high-secreting cells (>100 molecules/s) after 48 hours of stimulation (18.7% vs. 27.8%, for fresh and frozen cells respectively), not present after a 24-hour stimulation (35.1 vs 33.0%, for fresh and frozen cells respectively), with the total frequency of TNF-α SC being different between the two-time points. Hence the cryopreservation led to an increased high secretor population over time. The average in-droplet concentrations also only displayed differences after 48 and 72 hours of stimulation, where frozen hPBMCs reached higher endpoint concentrations (4.99 vs 9.38 nM at 48 h and 4.59 vs 9.95 nM at 72 h for fresh and frozen cells respectively).

Low frequencies of IL-1β secreting cells were found in response to stimulation after 24 and 48 hours (**Figure 3C**). However, an influence of freezing was only observed after the 24-hour stimulation, with secretion dynamics and rate distributions showing a short-lived peak for fresh hPBMCs that was not observed in thawed and stimulated cells. This observation was again likely due to the additional presence of high IL-1β SC (>10 molecules/s) that appeared earlier in fresh cells (14.2% *vs.* 4.3%). However, the exact magnitude of their extent remains unclear as the fitting projects them at 36 h, which was not an assayed time point.

Contrary to LPS stimulations, the appearance of IL-2 SC was observed after anti-CD3/CD28 stimulation (**Figure 3D**). There was only a minor influence of cryopreservation on the activation of these cells, i.e. their percentage of secreting cells. No IL-2 SC were detected after 72 hours of stimulation, indicating that the secretion of IL-2 was only temporary in this stimulation. However, the fresh stimulated cells consistently reached higher endpoint concentrations than frozen cells for 1 h (6.8 vs 5.3 nM) and 24 h stimulations (7.3 *vs.* 6.7 nM). Additionally, a small but consistently larger high-secreting population was found in fresh cells which was reduced in thawed cells. The dynamics of this secretion looked similar for all conditions, with a slow start reaching a plateau but with a delayed start at the first time point. In IL-2 SC, we further observed a significant difference in the secretion rate distribution after one-hour stimulation (p = 0.009). In brief, fresh cells displayed a consistently higher relative population of high-secreting IL-2 SC at all stimulation times, a population that was decreased in thawed and stimulated cells.

In terms of IL-8 secretion, we again saw a strong increase in the percentage of SC within cryopreserved hPBMCs (**Figure 3E**), with higher secretion after 24- and 48-hour stimulations (1.7% *vs.* 6.5% and 0.8% *vs.* 1.6%, fresh vs. frozen cells respectively). This was also apparent in the overtime analysis of the measured in-droplet concentrations. The secretion rate distributions of single SC also displayed changes, where significant differences towards higher secretion rates were found in the frozen sample for both time points (p < 0.0001), a change that was also present in the proportion of high-secreting cells (4^th^ panel). While they were mostly absent in fresh cells, freezing led to the appearance of up to 8.7% high-secreting SC. Additionally, a notable discrepancy in the distribution of secretion rates was already evident after one hour (p = 0.0001), and this finding was not due to the presence of additional high-secreting SC.

As expected, stimulation with anti-CD3/CD28 antibodies also resulted in a strong IFN-γ response (**Figure 3F**). IFN-γ SC were detected at all measured time points, with a slight increase for fresh cells after 24- and 48-hours of stimulation (5.4% *vs.* 4.3% and 12.5% *vs.* 9.5%). This did not generally translate into differences in measured in-droplet IFN-γ concentrations and secretion dynamics. Most interestingly, for the secretion rate distributions, there were significant differences for all measurements except the one-hour time point with a general increase of secretion rates in frozen cells, which is not attributable to additional high secreting cells (>100 molecules/s), but rather a shift of the whole population.

To summarize, we observed a heterogenous response after anti-CD3/CD28 stimulation. Apart from IL-2 and IL-8, a general trend towards reduced frequencies of secreting cells in thawed hPBMCs was observed. However, this decrease did not necessarily translate into decreased amounts of the secreted cytokine, indicating that the activation of cells and the secreted amount of cytokine are influenced differently by cryopreservation.

### Cryopreservation-induced alterations in specific polyfunctional subpopulations

**Figure 4:**
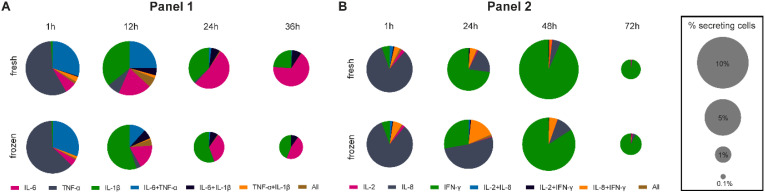
Pie charts representing the co-secreting populations over time of measured IL-6, TNF-α, IL-1β for LPS **(A)** and IL-2, IL-8 and IFN-γ for anti-CD3/CD28 **(B)** stimulations of fresh and cryopreserved hPBMCs. The number of cells in each bin was normalized to all detected SC per measurement in a single panel and the size of the pie chart was adjusted for the overall frequency of detected SC with a square root scaling factor for diameter. For comparison, the legend indicated the pie chart sizes for 10, 5, 1 and 0.1% SC. Stimulation times were identical to **Figures 2**, **3**.

In our measurements, we organized the cytokines into panels of three cytokines. Hence, the multiplexed single-cell measurements inherently allowed for the identification of polyfunctional cytokine responses in this study, corresponding to the simultaneous secretion of more than one secreted cytokine per cell. Since we were interested in how the cryopreservation process influences different polyfunctional subpopulations in response to stimulants, we analyzed our single-cell data in this regard. **Figure 4** shows the normalized polyfunctional response displayed within LPS- (A, 1^st^ panel) and anti-CD3/CD28-stimulated (B, 2^nd^ panel) fresh and thawed hPBMCs over the used incubation times.

The early response to LPS stimulation was mainly TNF-α dominated in both fresh and frozen conditions (**Figure 4A**), with most cells secreting TNF-α alone (57.2% *vs.* 62.2% for fresh and frozen cells, respectively), or TNF-α and IL-6 together (29.8% *vs.* 30.6%). 12-hour stimulations saw the appearance of IL-1β secretion, with a similar number of IL-1β SC between fresh and frozen but a higher relative frequency in frozen cells (35.8% *vs.* 55.0%) due to the reduction of IL-6 + TNF-α SC (24.8% *vs.* 11.7%) in frozen samples. The trend towards more relative IL-1β secretion in frozen cells was consistent for longer stimulations (36.8% *vs.* 55.3% and 23.7% *vs.* 43.6%, for 24- and 36-hour stimulations, respectively) alongside the decrease in frequency of IL-6 SC in the frozen sample (52.2% *vs.* 33.6% and 66.5% *vs.* 46.0%, for 24- and 36-hour stimulations, respectively). Hence, the polyfunctionality of cells secreting IL-1β was not affected by the cryopreservation if the cells secreted IL-1β alone or IL-1β + IL-6, whereas IL-6 secretion was affected (single as well as TNF-α co-secreting cells). Concerning IL-2, IL-8 and IFN-γ secretion following LPS stimulation, IL-8 was detected for all measured time points (**Supplementary Figure S3B**), with the complete absence of IL-2 and IFN-γ secretion, so no polyfunctional cells were detected for these measurements.

Differential polycytokine secretion was detected in response to anti-CD3/CD28 stimulation (**Figure 4B**). We primarily observed polyfunctionality in the panel measuring IL-2, IL-8 and IFN-γ. In the early response (1 hour), only minor differences between fresh and frozen cells were apparent, with most of the SC secreted either IL-8 alone (82.3% *vs.* 83.0%, for fresh and frozen cells respectively) or together with IFN-y (6.4% *vs.* 4.0%). However, 24-hour stimulation saw a distinct difference between fresh and frozen due to the 5-fold increase in the frequency of frozen cells secreting IL-8 only (19.5% vs. 51.8%) as well as polyfunctional cells secreting IL-8 + IFN-γ (5.3% *vs.* 16.9%). This increase coincided with a reduction of cells secreting IFN-γ alone (72.9% *vs.* 27.8%). Interestingly, those differences resolved at later time points as the majority of secretion was driven by IFN-γ (93.4% *vs.* 84.2%) but could still be seen at 48 hours for polyfunctional cells secreting IL-8 + IFN-γ (1.6% *vs.* 5.3%). In terms of secreted IL-1β, IL-6, and TNF-α secretion following anti-CD3/CD28 stimulation, only small differences were apparent, with minor alterations of the secreting cell populations after the 12-hour stimulation (**Supplementary Figure S3A**). In conclusion, polyfunctionalities were affected by cryopreservation and cytokine-specific as changes in polyfunctionalities were linked to alterations in IL-6 or IL-8 SC behavior.

### Intracellular cytokine staining highlighted an impact on cytokine expression in cryopreserved T cells and a disconnect between cytokine secretion and expression following anti-CD3/CD28 stimulation

To identify the SC that were most affected by cryopreservation, we performed an intracellular cytokine staining (ICS) combined with flow cytometry at selected time points. Due to the presence of all assayed cytokines, we measured short (1 hour) and longer (24 hours) responses in freshly isolated and cryopreserved hPBMCs stimulated with anti-CD3/CD28 antibodies.

The flow cytometry panel allowed the identification of the hPBMCs composition for each stimulatory condition and incubation time (**Supplementary Figure S4**). Over all conditions, the isolated hPBMCs from a buffy coat were composed on average of 60.7 to 81.7% T cells, 2.8-4.0% B cells, 4.0-6.17% NK cells and 0.1-8.95% CD14^+^ monocytes. The composition was similar between freshly isolated and frozen cells across all time points and stimulants, except for CD14^+^ monocytes. Firstly, their coefficient of variation between fresh and thawed hPBMCs was 30% after 1 h incubation and 17% after 24 hours. Additionally, CD14^+^ cells disappeared after 24 h stimulation with anti-CD3/CD28 stimulation in both fresh and frozen cells.

**Figure 5:**
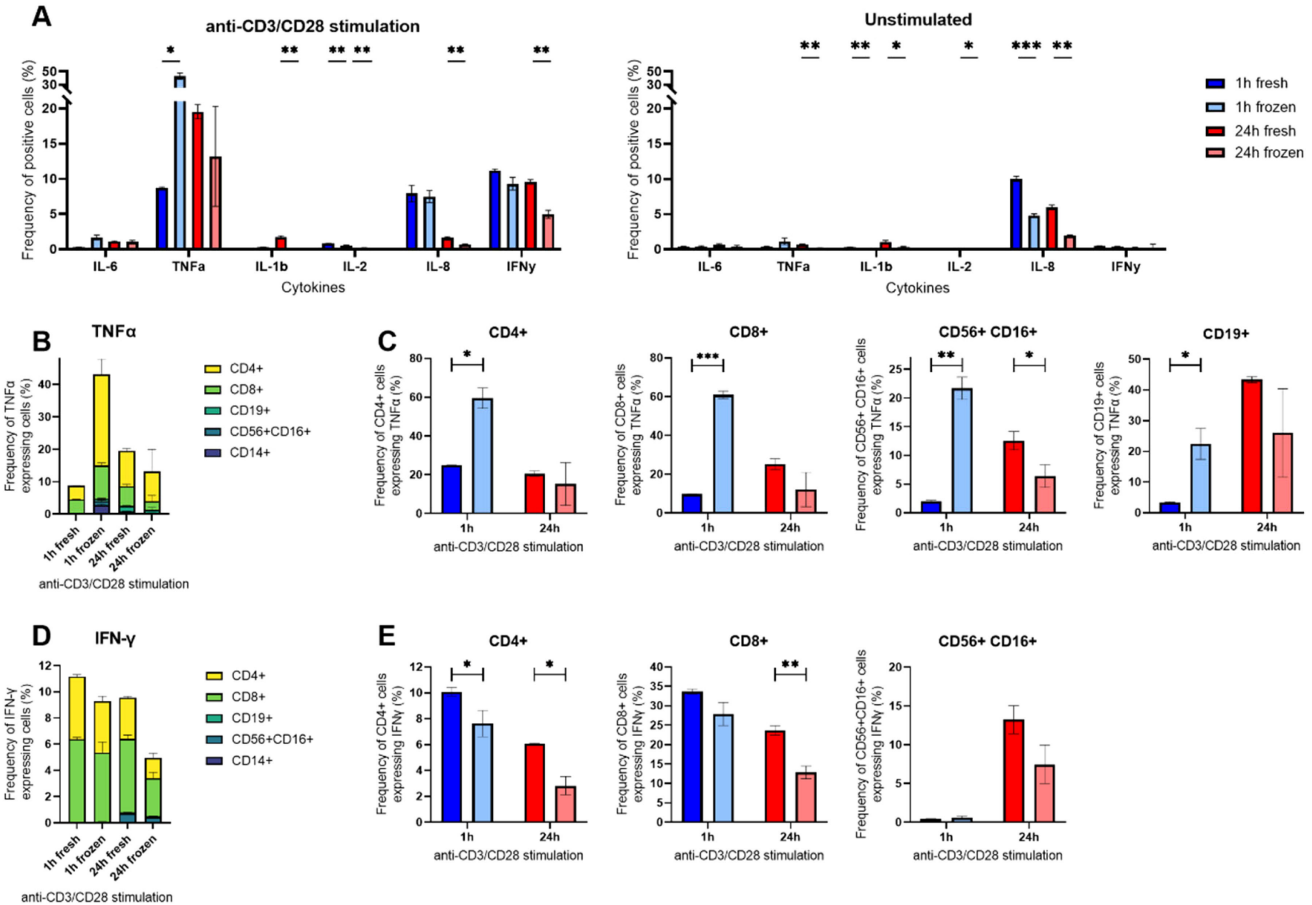
Intracellular cytokine staining (ICS) of anti-CD3/CD28-stimulated hPBMCs in flow cytometry. **(A)** Percentages of anti-CD3/CD28 stimulated or unstimulated hPBMCs identified as positive for measured IL-6, TNF-α, IL-1β, IL-8, IL-2 and IFN-γ after ICS gated on all alive single cells. **(B)** Percentages of TNF-α positive cells among all alive cells grouped by cell type. **(C)** Percentages of TNF-α positive cells within CD4^+^, CD8^+^, CD56^+^ CD16^+^ and CD19^+^ subpopulations at 1 h (blue) or 24 h (red) in cryopreserved (light shade) or fresh cells (bright shade). **(D)** Percentages of IFN-γ positive cells among all alive cells grouped by cell type expressing the cytokine. **(E)** Percentages IFN-γ positive cells within CD3^+^ CD4^+^, CD3^+^ CD8^+^ and CD3- CD56^+^ CD16^+^ subpopulations at 1 h (blue) or 24 h (red) in cryopreserved (light shade) or fresh cells (bright shade). Bar charts depict the mean frequency ± SD (n=3), and statistical differences were assessed using a multiple unpaired Welch t-test with alpha 0.05 (Holm-Šídák method). p-values were denoted as follows: * < 0.05, ** < 0.01, *** < 0.001, **** < 0.0001.

Next, we analyzed the cells for their cytokine expression using ICS (**Figure 5A**). The most notable difference was observed for TNF-α at 1 h, where 8.7% of fresh cells were positive compared to 43.1% in cryopreserved cells. Looking into the TNF-α positive cells, the impacted cells were mostly CD4^+^ and CD8^+^ T cells. These cells accounted for 8.4% of all positive cells in freshly isolated hPBMCs (4.2% CD4^+^ and 4.3% CD8^+^) and accounted for 38.4% in cryopreserved cells (28.1% CD4^+^ and 10.2% CD8^+^, **Figure 5B**). Within the population of T cells, we observed a 2.5-fold increase in CD4^+^ cells positive for TNF-α after 1 h stimulation with anti-CD3/CD28 antibodies (24.8% to 59.5%), and a 6-fold increase in CD8^+^ positives (9.7% to 60.9%, **Figure 5C**). Expanding to all the lymphocytes after 1 h stimulation for TNF-α, we also noticed a 6-fold difference for CD19^+^ cells (3.3% to 22.5%) and a 10-fold increase for CD56^+^ CD16^+^ cells (2.0% to 21.7%). Interestingly, these differences completely disappeared over time. Of note, this shift was not visible in the measurement of TNF-α SC in the microfluidic experiment (**Figure 3**).

Furthermore, we observed a distinct difference between fresh and frozen considering IFN-γ. We observed a reduction of IFN-γ^+^ cells after cryopreservation and 24 h stimulation with anti-CD3/CD28 antibodies, from 9.6% IFN-γ^+^ cells within fresh to 5.0% when cryopreserved (**Figure 5D**). This reduction was again attributed to T cells, with a 2-fold decrease in CD4^+^ IFN-γ^+^ cells (6.1% to 2.8%) and a 2-fold decrease in CD8^+^ IFN-γ^+^ cells (23.6% to 12.8%), respectively. Interestingly, the frequencies of IFN-γ^+^ CD56^+^ CD16^+^ cells after 24 h stimulation were not statistically different between the two conditions, indicating that the bulk activation of NK cells was not affected by the cryopreservation.

Surprisingly, we observed a peak in IL-1β expression in T cells after 24 h stimulation with anti-CD3/CD28 antibodies in freshly isolated cells (**Figure 5E**), a peak we also found in the secreting cells (**Figure 3**). This effect was present in freshly isolated T cells no matter if the cells were stimulated or not (**Supplementary Figure S5**). Concerning IL-8, the cryopreservation did not affect its expression in the stimulated cells, but greatly affected unstimulated cells (**Supplementary Figure S5**). As expected, the expression of this cytokine was mostly found in CD14^+^ cells.

### ICS on LPS-stimulated cells confirmed decreased expression of IL-6 and IL-8 SC after cryopreservation but displayed differences in IL-1β that were not visible in the secretion assay

**Figure 6:**
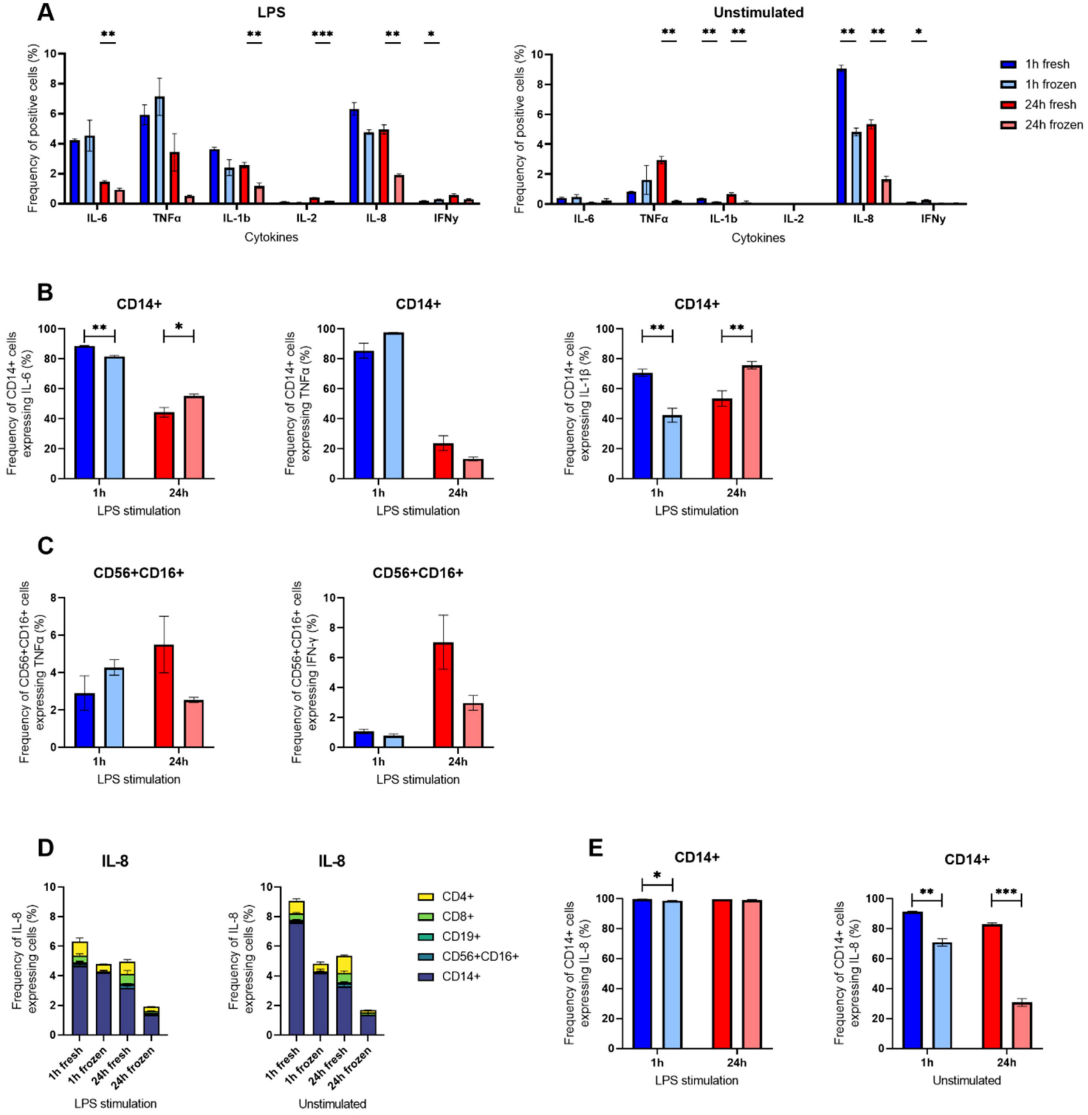
Intracellular cytokine staining of LPS-stimulated hPBMCs. **(A)** Percentages of LPS-stimulated or unstimulated hPBMCs identified as positive for measured IL-6, TNF-α, IL-1β, IL-8, IL-2 and IFN-γ after ICS in flow cytometry gated on all alive single cells. **(B)** Percentages of CD14^+^ positive cells for IL-6, TNF-α and IL-1β following 1 h (blue) or 24 h (red) stimulation or not with LPS in cryopreserved (light shade) or freshly isolated cells (bright shade). **(C)** Percentages of CD3- CD56^+^ CD16^+^ positive cells for TNF-α and IFN-γ following 1 h (blue) or 24 h (red) stimulation or not with LPS in cryopreserved (light shade) or freshly isolated cells (bright shade). **(D)** Percentages of IL-8 positive cells among all alive cells grouped by cell type. **(E)** Percentages of CD14^+^ positive cells for IL-8 following 1 h (blue) or 24 h (red) stimulation with LPS or unstimulated in cryopreserved (light shade) or freshly isolated cells (bright shade). Bar charts depict the mean of frequencies ± SD (n=3), and statistical differences were assessed using a multiple unpaired Welch t-test with alpha 0.05 (Holm-Šídák method). p-values were denoted as follows: * < 0.05, ** < 0.01, *** < 0.001, **** < 0.0001.

To further assess the effects of cryopreservation in innate immune cells, ICS was also performed with LPS-stimulated fresh and frozen cells (**Figure 6**). Generally, the cryopreservation did not affect the cytokine expression after 1 h stimulation but had a long-term effect for IL-6, IL-1β, IL-2, and IL-8, where the frequencies were lower in the cryopreserved condition (**Figure 6A**). Unsurprisingly, following LPS stimulation, the main cells expressing the cytokines were CD14^+^ for IL-6, TNF-α, IL-1β and IL-8 whereas CD56^+^ CD16^+^ secreted TNF-α, IL-2 and IFN-y (**Supplementary Figure S6**).

Taking a closer look at monocytes, the CD14^+^ cells were affected similarly for IL-6 and IL-1β, displaying both a decrease following cryopreservation at 1 h (88.8% to 81.7% for IL-6 and 70.7% to 42.4% for IL-1β) followed by an increase at 24 h (44.3% to 55.4% for IL-6 and 53.6% to 75.8% for IL-1β) LPS stimulation. On the other hand, the percentage of TNF-α expressing CD14^+^ cells was not significantly different following cryopreservation (**Figure 6B**).

The cytokine most influenced by cryopreservation was IL-8 (**Figure 6D**). However, this effect was particularly present in unstimulated control cells (9.1% to 4.8% at 1 h, and 5.3% to 1.7% at 24 h), but to a lesser extent in LPS-stimulated cells (6.3% to 4.8% at 1 h, and 4.9% to 1.9% at 24 h). Focusing on CD14^+^ cells, a similar observation could be made where the difference of IL-8 expressing CD14^+^ was striking between freshly isolated and cryopreserved cells without any stimulation (91.26% to 70.76% at 1 h and 82.96% to 30.66% at 24 h) but masked following LPS stimulation as nearly all CD14^+^ were positive for IL-8 (99.73% to 98.63% at 1 h and 99.6% to 99.16% at 24 h).

NK cells were not considerably affected by the cryopreservation, as their frequencies of TNF-α and IFN-γ expressing cells were not significantly different between freshly isolated and cryopreserved CD56^+^ CD16^+^ cells (**Figure 6C**). Interestingly, we also observed a small population of CD56^+^ CD16^+^ expressing IL-2 and IFN-γ (**Supplementary Figure S6**), two cytokines we did not find secreted in the microfluidic experiments.

Overall, monocytes were more affected than NK cells by cryopreservation and LPS stimulation, specifically IL-6, IL-8 and IL-1β. While IL-6 and IL-8 were also strongly affected, the other differences could not be corroborated in the secretion assay (**Figure 2**).

## Discussion and outlook

Cryopreservation of immune cells is a widely adapted technique in many research laboratories and also holds importance in clinical settings to ship and store samples (14). If cytokine readouts of cryopreserved cell are to be used as readouts, it has to be established first how secretion is influenced by cryopreservation. Therefore, it seems of utmost importance to quantify differences in the cytokine secretion behavior after cryopreservation as alterations in cytokine expression and secretion have been well-described using traditional methods such as ELISA or ICS (21–23), and we add data for different cellular subpopulations, cytokines, stimulation times and stimulants on a functional single-cell level. Concretely, the objective of this study was to investigate the changes in the secretion behavior of specific cytokines following stimulation in a dynamic and direct functional manner and to decipher the potential loss in frequencies, subpopulations and cellular output separately. In addition to lower or higher cytokine secretion, this approach also allowed visualizing potential differences in dynamics and specific cytokines, or cell types affected most by cryopreservation. Furthermore, these results might help to compare samples from frozen, thawed and stimulated hPBMCs with fresh stimulated samples, which is of special interest when small numbers of rare disease samples are collected for later analysis.

Following LPS stimulation, our flow cytometry data identified mostly CD14^+^ monocytes as the main source of cytokine secretion for TNF-α and IL-1β (**Supplementary Figure S6**), especially early on. Here, in contrary to our initial expectations, we observed only minor effects of cryopreservation on the secretion behavior of TNF-α and IL-1β (**Figures 2A, C**). The secretion by mainly one cell subpopulation could be the reason why secretion showed little susceptibility to cryopreservation (33). While TNF-α showed a high early secretion with rapid decrease, IL-1β had a distinct maximum of secreting cells around 12 h. However, droplet single-cell analysis revealed slight differences in secretion rate distributions for TNF-α, mostly in the dynamics where fresh cells were secreting generally more rapidly than thawed cells after stimulation. This kinetic disconnect could be explained by faster TNF-α responses and a slower IL-1β response after stimulation (34). Another study found increased IL-1β concentrations in the supernatant after LPS stimulation for cryopreserved cells (16), which we could not confirm in our study, and additional studies will be necessary to confirm the conflicting results found in the literature.

On the other hand, IL-6 and IL-8 secretion by CD14^+^ cells after LPS stimulation were strongly affected by cryopreservation, with a lower number of SC and alterations in secretion rate distributions across all measured incubation times. Except for the 1-hour timepoint for IL-6, the number of SC was lowered by about the same amount for all incubation times and both cytokines (-2.5% for IL-6 and -4.5% for IL-8, respectively, **Figures 2A, D**), meaning these cells did not recover their ability to secrete over time. However, while both high- and low-secreting IL-6 SC were affected, the decrease was stronger in high-IL-8 SC. Interestingly, the polyfunctionality of cells secreting these cytokines also seemed affected, but only in some specific cases (IL-6 + TNF-α) as it was not always the case (IL-6 + IL-1β). These findings are interesting considering that some reports have shown that the cytokines response profiles were lower in cryopreserved monocytes for some cytokines (35) while others highlighted a reduction in the numbers of the monocyte population (14) or within monocyte subsets (36) following freezing. Hence, while the variation of the CD14^+^ cells frequency across all conditions makes it more difficult to compare secretion differences, it could be explained by a differential susceptibility of the monocyte subsets to freezing.

To examine changes in different cell types, we also stimulated fresh and thawed hPBMCs with anti-CD3/CD28 antibodies, where secretion was mostly found in T cells. While only little secretion of IL-6 was detected and only small changes for TNF-α and IL-2, the secretion of IL-1β, IL-8, and IFN-γ was differently affected by cryopreservation (**Figure 3**). While TNF-α secretion remained similar, the frequency of positive cells in the ICS dramatically increased in cryopreserved cells after 1 hour (**Figure 5**). This result highlights a potential disconnect between the expression and secretion of TNF-α within T cells. Cryopreservation appeared to make these cells more susceptible to stimulation in the short term (1 hour), but this observation did not translate into higher secretion as there were only small changes in the number of SC and lightly shifted secretion rate distributions of individual cells (**Figure 3B**). This observation supports two previous studies that highlighted increases in TNF-α expression on the transcriptional level, which did not translate into a difference in protein production (36, 37). Then, IL-1β had a higher frequency in fresh cells after 24 h stimulation, with a short-lived peak of cells found in ICS and the secretion assay, which was probably due to its presence within T cells in the presence of IL-2, as it has already been described in the literature (38).

The cytokine IL-8 showed distinct behaviors following both stimulations, after anti-CD3/CD28 stimulation we saw increased frequencies of SC when cells were cryopreserved (**Figure 3D**) whereas we observed a reduction when cryopreserved cells were stimulated with LPS (**Figure 2D**). This difference in susceptibility was however not observed within the ICS experiments, where the cryopreservation led to reduced frequencies in unstimulated conditions that were rescued following both stimulants in early time points. It is important to note here the disappearance of CD14^+^ monocytes after 24 h stimulation with anti-CD3/CD28 antibodies, which was also likely due to activated T cells inducing their apoptosis (39, 40). Hence, this distinct response could be due to the heterogeneity of the CD14^+^ population or expression changes that did not result in secretion changes. Furthermore, the co-secretion analysis revealed an increase in the frequency of cells secreting IL-8 alone as well as cells secreting both IL-8 and IFN-γ after cryopreservation (**Figure 4B**).

Lastly, the IFN-γ response following anti-CD3/CD28 stimulation was only minimally affected by cryopreservation, particularly at longer incubation times in terms of cytokine output per cell (**Figure 3F**) or in the frequency of positive CD4 and CD8 T cells (**Figure 5D**). This reduced response has been previously described for CD3^+^ CD4^+^ cells and effects of cryopreservation were also observed in polyfunctional cells expressing additional IL-2 and/or TNF-α (41). Our observation on polyfunctionality also showed a lower frequency of cells secreting IFN-γ alone and an increased frequency of cells co-secreting IFN-γ and IL-8, which were resolved by longer incubation times. Hence, the effects on polyfunctionality seem to be linked to IL-8 alterations and a different cytokine panel could have helped to corroborate these observations. Additionally, there was again a disconnect between IFN-γ expression and secretion. Indeed, the frequency of cells secreting IFN-γ was low in early stimulation times (**Figure 3F**), whereas it was already seen at the 24-hour level in the ICS (**Figure 5D**). Differences in IFN-γ have been reported if the cells were activated through TCR, with a peak in frequency at 6 hours, or by cytokine cocktails with a peak at 36 h, similar to our observations as well (42). Besides, IFN-γ expression was found in NK cells after 24 hours of stimulation with anti-CD3/CD28 antibodies, showing their indirect activation during the bulk stimulation as already described (43), which did not seem to be affected by cryopreservation. Finally, IL-2 secretion was generally low in all the conducted experiments, making it difficult to conclude the influences of cryopreservation on the production and secretion of this cytokine.

An often-observed result was the disconnect between the intracellular cytokine staining and the single-cell secretion measurement methods. For example, TNF-α or IFN-γ showed a clear disconnect between expression and secretion. As the microfluidic method measured SC whereas ICS measured intracellular cytokine presence, this could imply that even if cryopreservation led to changes in expression, these changes did not always lead to phenotypical changes in secretion. Hence, additional studies would be needed to identify how and which molecular mechanisms controlling cytokine secretion are affected by cryopreservation. Additionally, disconnects in readouts highlight the importance of the methods used, where it is possible to differentiate between expression and physiological secretion which might be best analyzed directly.

The limitations of the performed study include the limited comparability between the different stimulation experiments due to the anonymized blood donors. The influence of stimulation time and cryopreservation were described within every experiment between the same donor and were therefore comparable. Inherently limiting, the single-cell droplet measurements always include the potential bias of autocrine or the absence of paracrine effects. However, incubation in bulk should, at least partly, mitigate the absence of paracrine signaling in the droplets, and we have shown before that if autocrine effects occur, they do not occur on a systemic or population level (27). In general, different cell subpopulations are differently affected by cryopreservation, and the impact of this procedure on the results will be inherently influenced by the chosen stimulation method as well as analysis method. Finally, a more extensive study incorporating diverse methods, and a broader range of donors is essential to thoroughly comprehend the effects of cryopreservation and donor variability on cytokine secretion behavior. Investigating polyfunctionality will be particularly challenging, as it inherently depends on the specific combination of cytokines being measured.

In summary, we demonstrated the use-case of novel single-cell cytokine assays as well as the need to combine them with traditional methods, such as ICS, to understand the differences between secretion and expression. Together, they offer an in-depth insight into the biological behavior of immune cells and the changes introduced by cryopreservation and might help to generate new understandings of the observed differences and inform new applications in clinical settings about the potential use of frozen hPBMCs.

## Data Availability

The original contributions presented in the study are included in the article/**Supplementary Material**. Further inquiries can be directed to the corresponding author.
