# Supplementary material for: The impact of cryopreservation on cytokine secretion and polyfunctionality in human PBMCs: a comparative study
Source: Front Immunol. 2024 Oct 7;15:1478311. doi: 10.3389/fimmu.2024.1478311 (PMC11491348; doi:10.3389/fimmu.2024.1478311)
Supplement: Supplementary file 1 [file Table1.pdf]

Supporting information

The Impact of Cryopreservation on Cytokine Secretion and Polyfunctionality in Human PBMCs: A Comparative Study

Aline Linder[1*], Kevin Portmann[1*] and Klaus Eyer[1,2#]

[1]Laboratory for Functional Immune Repertoire Analysis, Institute of Pharmaceutical Sciences, Department of Chemistry and Applied Biosciences, ETH Zürich, 8093 Zürich, Switzerland.

[2]Department of Biomedicine, Aarhus University, The Skou Building Høegh-Guldbergs Gade 10, DK-8000 Aarhus C, Denmark.

# Correspondence: eyerk@biomed.au.dk

* These authors contributed equally to this work and share first authorship.

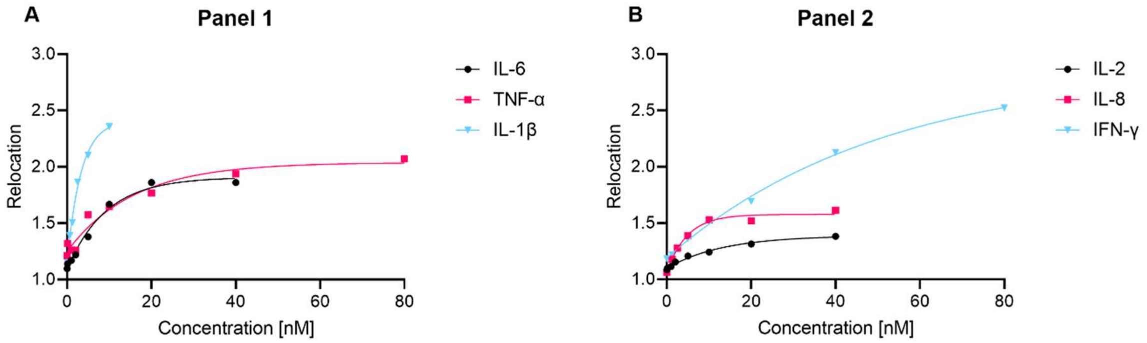

S1 Figure. Calibration curves for the used cytokine panels. Measured fluorescence relocation against the in-droplet cytokine concentration of IL-6, TNF-α, IL-1β (A) and IL-2, IL-8, IFN-γ (B). Data was fitted with non-linear one phase association curve fits (GraphPad Prism). Fits were used to calculate concentration out of relocation values during cellular measurements. The points were relocation dropped again due to the Hook effect (above the maximal measured relocation) were excluded from the analysis.

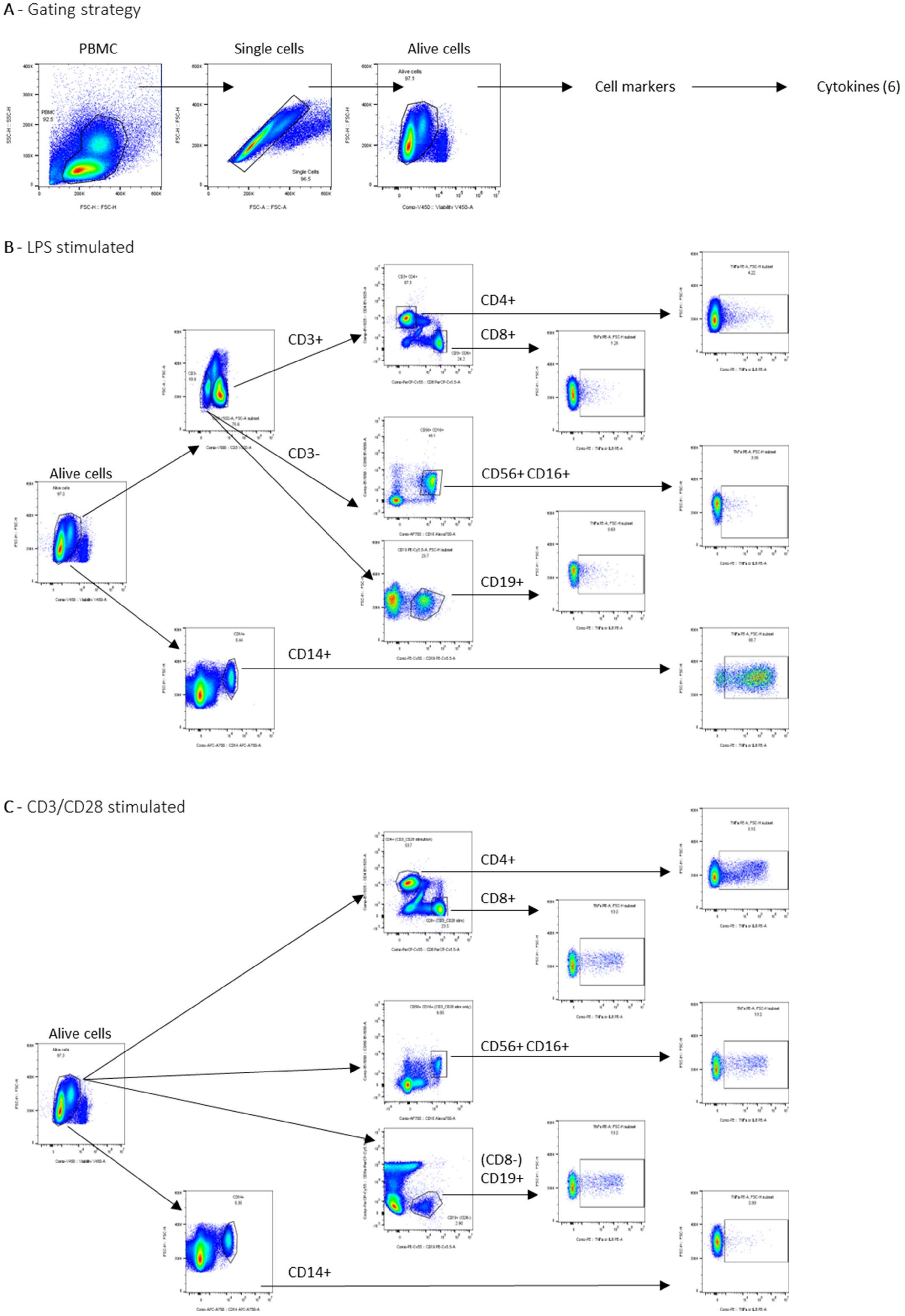
A - Gating strategy
PBMC
Single cells
Alive cells
Cell markers
Cytokines (6)
B - LPS stimulated
Alive cells
CD3+
CD3-
CD4+
CD8+
CD56+ CD16+
CD19+
CD14+
C - CD3/CD28 stimulated
Alive cells
CD4+
CD8+
CD56+ CD16+
(CD8-)
CD19+
CD14+

D - Cytokines

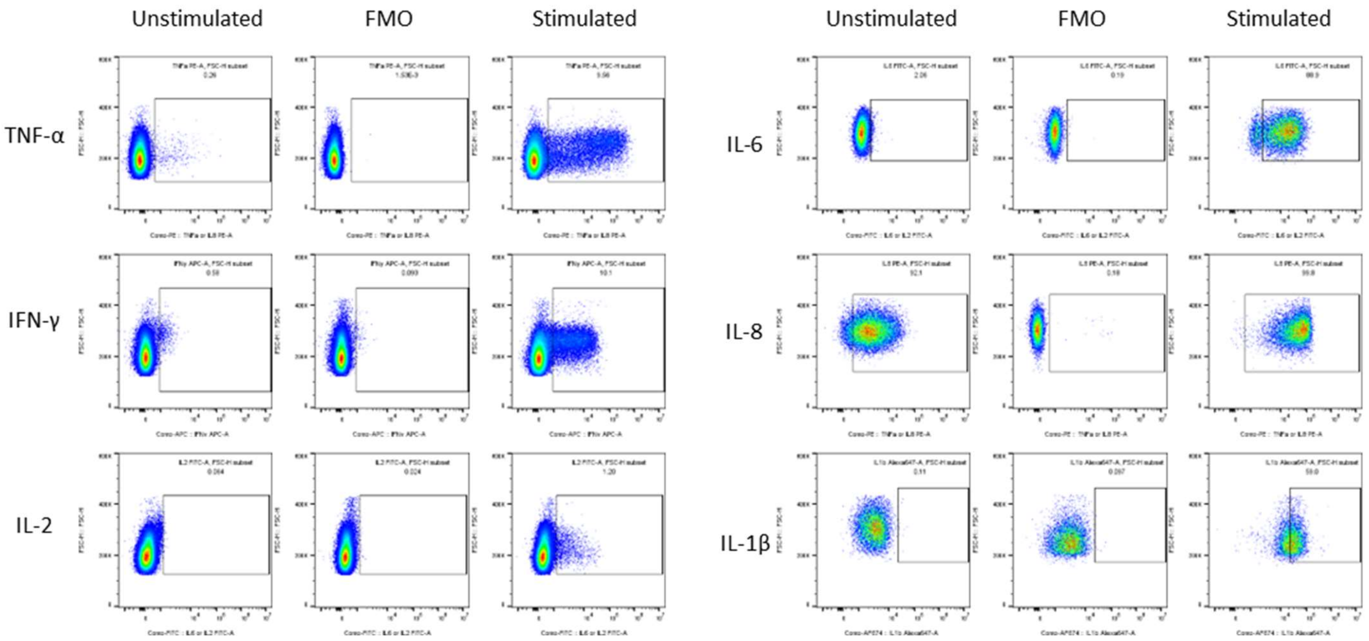

S2 Figure: Gating strategy for the identification of the cytokine producing cells. hPBMCs from a buffy coat were isolated or thawed, stimulated with (B) LPS or (C) anti-CD3/CD28 antibodies for 1h or 24h, then brefeldin A and GolgiStop were added for 4h. Surface and cytokine stainings were then performed (See antibodies used in table 1). The cells were run on a CytoFLEX S and analyzed using the FlowJo software version 10.8.1. The flow cytometer dot plots illustrate the strategy for the identification of the cytokine producing cells. hPBMCs were identified through SSC-H and FSC-H, single-cells were discerned from aggregates through FSC-H and FSC-A and alive cells were selected as negative for the viability dye. Alive cells were further separated into monocytes (CD14+), T cells (CD3+ CD4+ or CD3+ CD8+), NK cells (CD3- CD56+ CD16+) and B cells (CD3- CD19+). For the cells stimulated with an anti-CD3/CD28 antibodies, contaminating CD8+ T cells were removed to identify the B cells (CD3- CD19+). The frequency of cytokine producing cells were then assessed within each cell population, for each cytokine (IL-6, TNFα, IL-β, IL-2, IL-8 and IFN-γ). The FMO (Fluorescence minus one) controls were used for each fluorochrome to set the gates (D).

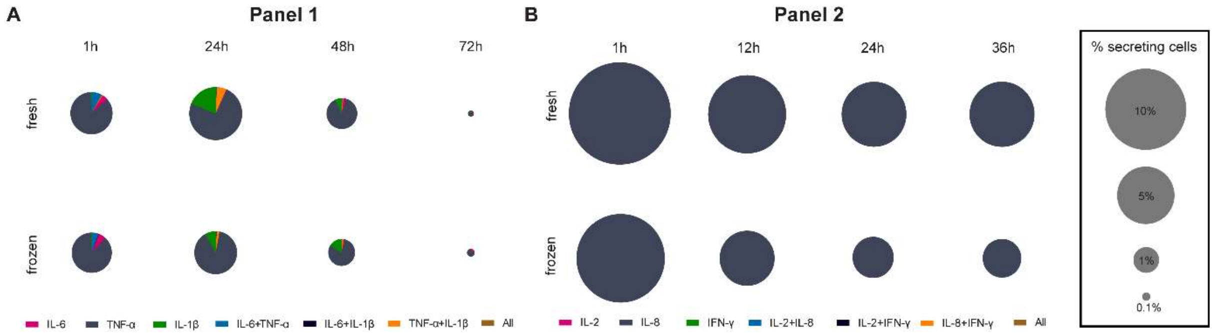

S3 Figure. Pie charts representing the co-secreting populations of measured IL-6, TNFα, IL-1β and IL-2, IL-8 and IFN-γ for different stimulations and fresh/frozen hPBMCs. Shown are pie charts representing the different secreting cell populations of the measured cytokines. The panels measured IL-6, TNFα, IL-1β (A) and IL-2, IL-8, IFN-γ (B) for freshly isolated and frozen hPBMCs. Fresh or frozen hPBMCs were stimulated with anti-CD3/CD28 antibodies (A) for 1, 24, 48, 72 hours or LPS (B) for 1, 12, 24, 36 hours and binned into the respective categories. The size of each pie chart is adjusted based on the maximal frequency of secreting cells detected in all measurements (**Fehler! Verweisquelle konnte nicht gefunden werden.**B, 48h fresh) with the following formula: $scaling\ factor = \sqrt{frequency}/\sqrt{frequency_{max}}$. For comparison a legend showing the pie chart size for 10, 5, 1 and 0.1% secreting cells is shown.

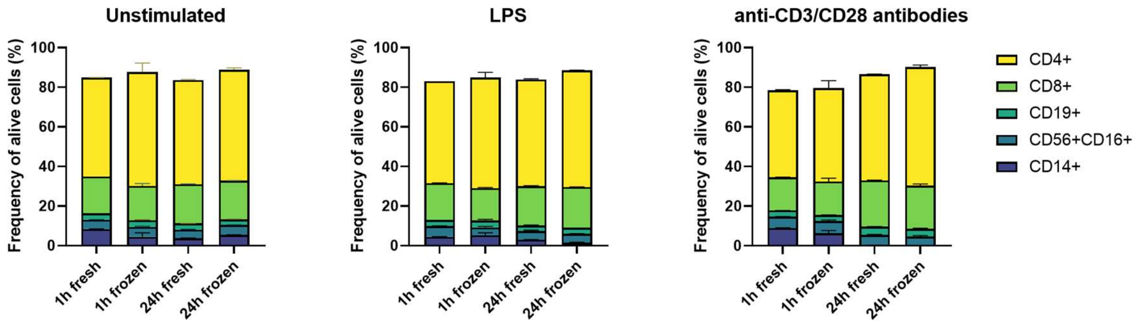

S4 Figure: Cell type proportions across the different timepoints and conditions, following (A) anti-CD3/CD28 stimulation, (B) LPS stimulation or (C) unstimulated. Note the loss of the CD14+ population after 24h stimulation with anti-CD3/CD28 antibodies. For each condition, the average frequency of the alive cells for the T cells (CD4+ and CD8+), B cells (CD19+), NK cells (CD56+CD16+) and monocytes (CD14+) are represented with the stacked bars with SD (n=3).

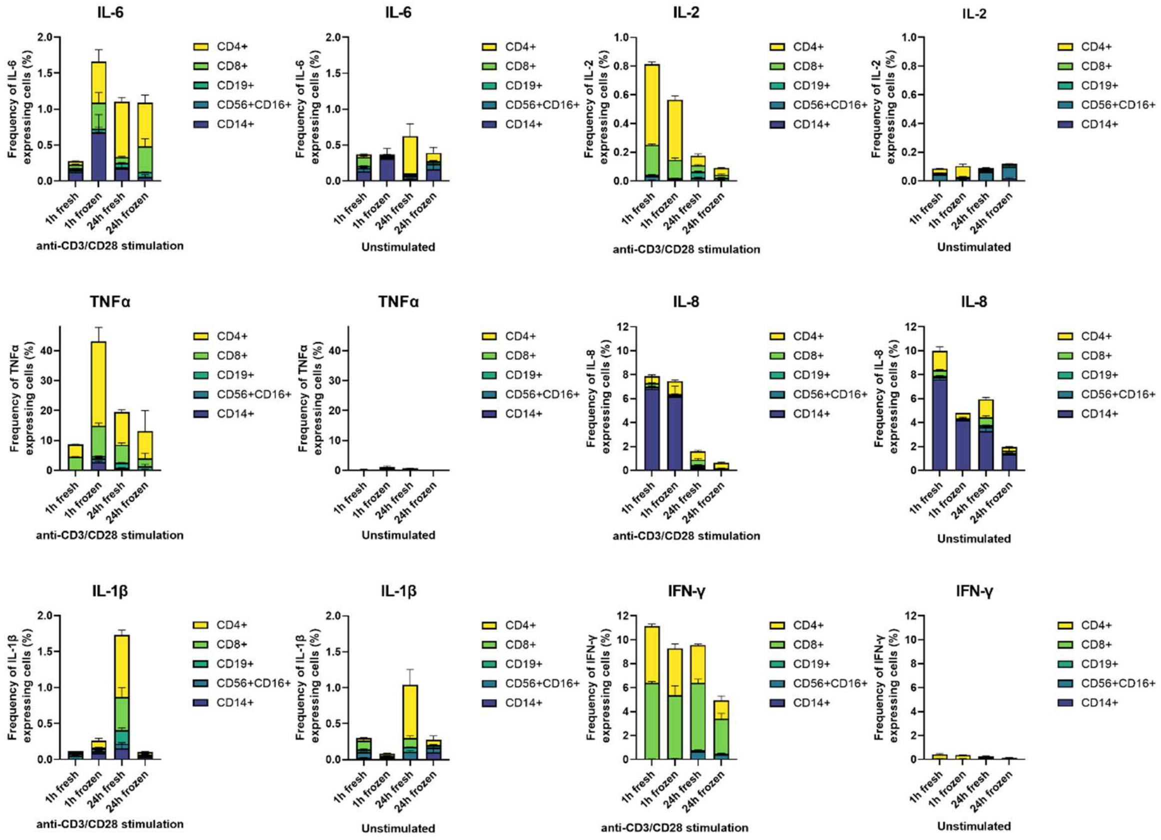

S5 Figure: Percentages of positive cells among alive cells with the identification of the cell type expressing the cytokine following anti-CD3/CD28 stimulation or without stimulant. Data represented as stacked bars with SD (n=3).

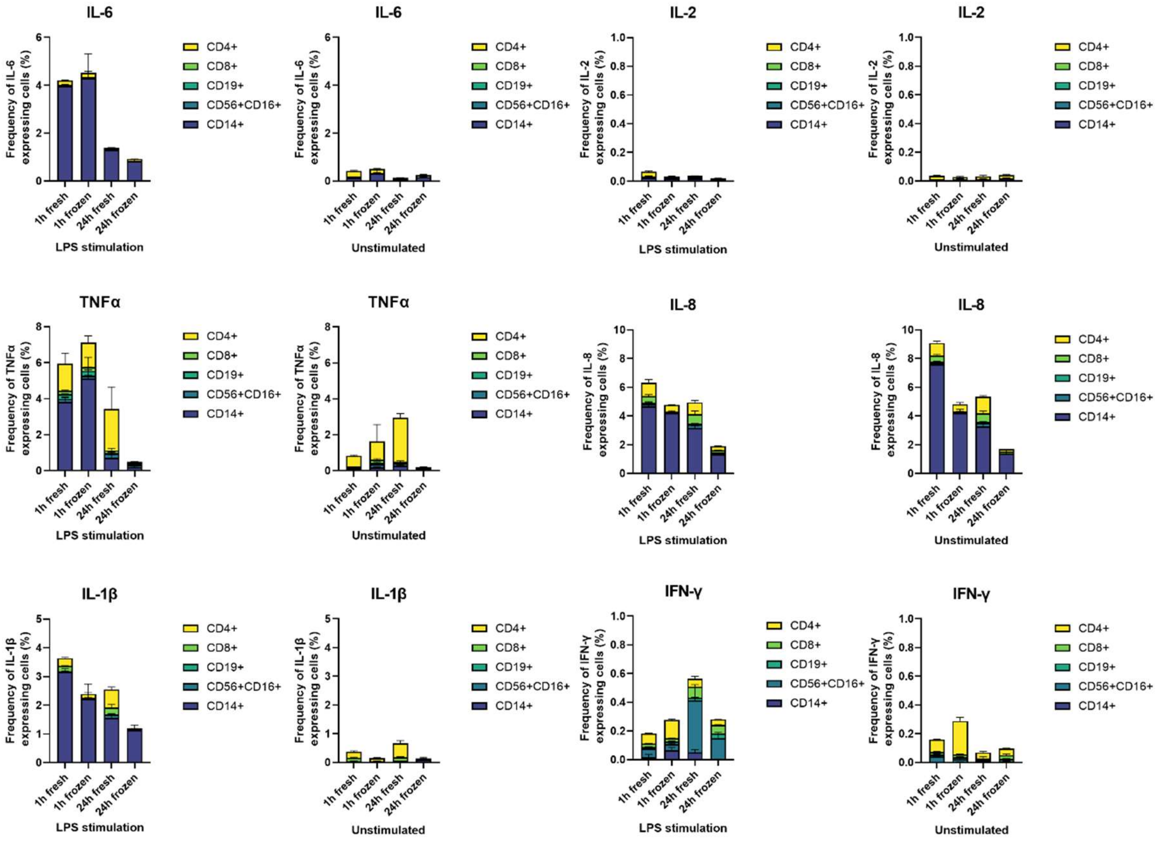

S6 Figure: Percentages of positive cells among alive cells with the identification of the cell type expressing the cytokine following LPS-stimulation or without stimulant. Data represented as stacked bars with SD (n=3).
